# Simple screening models for cognitive impairment in community settings: The IRIDE Cohort Study

**DOI:** 10.1111/ggi.14360

**Published:** 2022-02-20

**Authors:** Takumi Abe, Akihiko Kitamura, Mari Yamashita, Hunkyung Kim, Shuichi P. Obuchi, Tatsuro Ishizaki, Yoshinori Fujiwara, Shuichi Awata, Kenji Toba, IRIDE Cohort Study investigators

**Affiliations:** ^1^ Integrated Research Initiative for Living Well with Dementia Tokyo Metropolitan Institute of Gerontology Tokyo Japan; ^2^ Research Team for Social Participation and Community Health Tokyo Metropolitan Institute of Gerontology Tokyo Japan; ^3^ Research Team for Promoting Independence and Mental Health Tokyo Metropolitan Institute of Gerontology Tokyo Japan; ^4^ Human Care Research Team Tokyo Metropolitan Institute of Gerontology Tokyo Japan; ^5^ Tokyo Metropolitan Institute of Gerontology Tokyo Japan; ^6^ Tokyo Metropolitan Geriatric Hospital and Institute of Gerontology Tokyo Japan

**Keywords:** area under the curve, cognitive impairment, integrated discrimination improvement, net reclassification improvement, screening test

## Abstract

**Aim:**

Community settings often need simple screening, rather than detailed tests, to identify cognitive impairment. This study aimed to develop models to screen older adults with cognitive impairment.

**Methods:**

This study used data from the Integrated Research Initiative for Living Well with Dementia Cohort Study and included 5830 older adults. Individuals were considered cognitively impaired if their Mini‐Mental State Examination score was less than 24. Three screening models were developed: the simple model (age, sex, and education), the base model comprising 13 candidate variables available in the questionnaire, and the enhanced model, where grip strength and gait speed were added to the base model. We performed binary logistic regression analysis with stepwise backward elimination (*P* < 0.1 for retention in the model) to develop each model. Then, we calculated integer scores from coefficients to develop score‐based models. The area under the receiver operating characteristic curve (AUC) was used to evaluate discrimination.

**Results:**

Participants with cognitive impairment accounted for 4.0% (*n* = 233) of the total. The score‐based simple model comprised three variables (AUC = 0.72, sensitivity: 72%, specificity: 61%). The score‐based base model included nine variables (AUC = 0.76, sensitivity: 70%, specificity: 67%). The score‐based enhanced model comprised eight variables, including grip strength and gait speed (AUC = 0.79, sensitivity: 73%, specificity: 70%).

**Conclusions:**

This study developed three screening models with acceptable discriminant validity for cognitive impairment. These models comprised simple questionnaire‐based items and common physical performance measurements. These models could enable screening of older adults suspected of cognitive impairment without the need to conduct cognitive tests in community settings. **Geriatr Gerontol Int 2022; 22: 292–297**.

## Introduction

An increase in the number of people with dementia is a global trend, and it is estimated that in Japan over 20% of older adults will have dementia in 2030.^1^ Accordingly, the cabinet of Japan developed on the National Framework for Promotion of Dementia Policies in June 2019, discussing comprehensive strategies associated with dementia.^2^ This framework discusses the promotion of actions to delay the onset of dementia in cognitively healthy individuals and to identify individuals with cognitive impairment. Public sectors, especially stakeholders involved in health promotion activities, play a significant role in this context, as those without dementia are more likely to live in their community rather than in care settings such as nursing homes. The framework also discusses inclusiveness, implying that people should continue to live in a place where they are used to living, even if they have cognitive impairment or dementia. Receiving support from others could allow those with cognitive impairment to continue living in the community.^3^ Therefore, identification of those with cognitive impairment in community settings is important before they need to reside in a care facility.

Conducting a cognitive assessment in community settings as opposed to clinical settings is sometimes difficult—particularly the one‐to‐one interview method, as, for example, in disability prevention programs. This may be because there are insufficient staff with the expert knowledge required to administer cognitive tests. Since there are some cases where direct assessments are not suitable in community settings, alternative specialized methods are needed. Indeed, novel methods such as computer‐based^4^, ^5^ and physical performance‐based^6^, ^7^ assessments of cognitive functions have been developed. Such novel methods, however, have disadvantages. Some computer‐based assessments, which can involve programmed standardized cognitive tests, take more time to complete, and the installation of the computers needed for the tests might make these assessments less feasible in terms of cost. Physical performance‐based cognitive assessments often require specific equipment. These characteristics make it hard to conduct such assessments in community settings. Therefore, alternative methods that are suitable to assess cognitive function in community settings, such as simple screening tools for individuals with cognitive impairment, are required.

Risk prediction models for the onset of dementia have been developed. A Japanese longitudinal study showed that a risk prediction model comprising age, sex, and the Kihon Checklist, which is a standardized self‐reported questionnaire to assess functional abilities, has acceptable discriminant validity.^8^ Another study provided a useful risk chart to calculate the 10‐year risk of dementia: the chart was developed by combining age, history of stroke, subjective memory decline, and need for financial or medical assistance.^9^ These findings could be applied in community settings, since the risk model/chart comprises simple questions. Risk prediction is beneficial from the perspective of dementia prevention, and it is also important to assess individuals' current cognitive function. However, no such models are currently available for cognitive impairment screening.

This study aimed to develop models for cognitive impairment screening without using cognitive assessment tests and to evaluate their discrimination and calibration.

## Methods

### Participants

This study used data from the Integrated Research Initiative for Living Well with Dementia Cohort Study (IRIDE‐CS), which is an integrated study initiated by the Tokyo Metropolitan Geriatric Hospital and Institute of Gerontology. The IRIDE‐CS was launched in 2020 to provide evidence associated with cognitive impairment and dementia. The IRIDE‐CS comprises five cohort studies: the Otassha Kenshin Study; Takashimadaira Study; Septuagenarians, Octogenarians, Nonagenarians Investigation with Centenarians (SONIC) Study; Hatoyama Cohort Study; and Kusatsu Longitudinal Study on Aging. Participants in these five cohorts were aged 65 years or older, and there were no other common criteria. The characteristics of the five cohorts are shown in Table S1. Each cohort provides the IRIDE‐CS with data based on its data availability policy. The total sample size of the IRIDE‐CS was 7833; of these participants, 7719 completed cognitive tests (Otassha Study, 3289; Takashimadaira Study, 2019; SONIC Study, 567; Hatoyama Cohort, 723; and Kusatsu Longitudinal Study on Aging, 1121). The present study excluded data from the SONIC Study, which used different ways to assess physical function from the other four cohorts. The Takashimadaira Study included participants who took part in a door‐to‐door survey only (i.e., they did not participate in the field survey), and thus we excluded them from the present study (*n* = 667). The IRIDE‐CS was approved by the ethical committee of the Tokyo Metropolitan Institute of Gerontology.

### Outcome variable

Cognitive function was assessed by the Mini‐Mental State Examination (MMSE).^10^ The MMSE score ranges from 0 to 30; a higher score indicates higher levels of cognitive function. This study used the conventional cut‐off point (<24 points) for cognitive impairment.

### Candidate variables

Assuming that the models we are trying to develop are to be used in community settings, but not research and clinical settings, it would be rational to select common variables. We referred to previous reviews^11^, ^12^ and selected common factors that can be obtained from a questionnaire. In this procedure, we prioritized the selection of a single question, as it is not practical to use screening models that comprise many items in community settings. Physical function measurements, which are associated with cognitive function,^13^ are often used for assessments in disability prevention programs conducted in community settings.^14^ Thus, we considered adding those measurements.

We finally selected 15 variables. Thirteen variables were included as basic information: age (65–74, 75–84, ≥85 years), sex, body mass index (BMI) (<18.5, 18.5–24.9, ≥25.0 kg/m^2^), education (<9, 9–12, ≥12 years), history of hypertension, hyperlipidemia, diabetes, stroke, drinking (current, past, never), smoking (current, past, never), frequency of going outdoors (≤2 days/week, 3–6 days/week, every day), visual impairment (yes, no), and hearing impairment (yes, no). Variables for hypertension, hyperlipidemia, and diabetes were converted into three categories (never, under treatment, and non‐treatment), while those for stroke were divided into two categories (yes and no). Stroke often causes paralysis and is likely to have a large impact on health status compared with the other three diseases; hence, we did not handle the data regarding stroke in the same manner. As physical function measurements, we selected two common items: grip strength (low, normal/high), and gait speed (slow, normal/high). Low grip strength and slow gait speed were defined using the cut‐off point for frailty and sarcopenia: <28 kg for men and <18 kg for women, and <1.0 m/s for both sexes.^15^, ^16^

### Statistical analysis

Binary logistic regression analysis with stepwise backward elimination (*P* < 0.1 for retention in the model) was conducted to develop models for cognitive impairment screening. Since including physical function measurements as essential factors limits generalizability, this study yielded three models: the simple model, base model, and enhanced model. The simple model included three variables—age, sex, and education—to minimize the number of essential variables. The base model comprised basic information (i.e., the above‐listed 13 variables). For the enhanced model, the two physical function measures were added to the base model. The three models were adjusted for a dummy variable indicating each cohort study.

Discrimination was tested using the area under the receiver operating characteristic curve (AUC). The difference of AUC was tested using the DeLong test.^17^ The change in AUC strongly depends on the fundamental model, and thus we also calculated the continuous net reclassification improvement (NRI) and the integrated discrimination improvement (IDI) to assess the effect of adding new predictors.^18^ We used the bootstrapping method with 1000 resamples to calculate the 95% confidence interval (CI) for NRI and IDI using the Stata command “incrisk.”

We converted coefficients to integer scores in each model, improving the usability of the developed models. In this procedure for developing score‐based models, we adjusted the minimum regression coefficient to 1 (e.g., if the minimum regression coefficient was 0.4 in the model, each integer score was calculated by the β multiplied by 2.5).^19^ Since rounding generally causes a reduction of discrimination, we calculated the AUC of the score‐based models. Finally, we determined the sensitivity and specificity by the maximum of the Youden index (sensitivity + specificity − 1).

We needed to use both because the Hosmer‐Lemeshow test is not always accurate when the sample size is large.

All statistical analyses were conducted using Stata 16 (StataCorp, College Station, TX). Except for the stepwise backward elimination, statistical significance was set at *P* < 0.05.

## Results

After excluding participants with missing data (*n* = 655), this study included 5830 participants for analysis: the Otassha Kenshin Study, *n* = 3112; Takashimadaira Study, *n* = 1121; Kusatsu Longitudinal Study on Aging, *n* = 693; and Hatoyama Cohort Study, *n* = 904. Participants with cognitive impairment accounted for 4.0% (*n* = 233). Table 1 shows the characteristics of participants. The mean (standard deviation) MMSE scores of all participants and those with and without cognitive impairment were 28.1 (2.2), 28.4 (1.6), and 21.0 (2.7), respectively.

**Table 1:** Characteristics of participants

		All (*N* = 5830)	Participants without cognitive impairment (*N* = 5597)	Participants with cognitive impairment (*N* = 233)
	Category	*n* (%)	*n* (%)	*n* (%)
Age	65–74 years	3100 (53.2)	3044 (54.4)	56 (24.0)
	75–84 years	2501 (42.9)	2362 (42.2)	139 (59.7)
	≥85 years	229 (3.9)	191 (3.4)	38 (16.3)
Sex	Women	3864 (66.3)	3738 (66.8)	126 (54.1)
Body mass index	<18.5 kg/km^2^	441 (7.6)	413 (7.4)	28 (12.0)
	18.5–24.9 kg/km^2^	3983 (68.3)	3836 (68.5)	147 (63.1)
	≥25 kg/km^2^	1406 (24.1)	1348 (24.1)	58 (24.9)
Education	<9 years	217 (3.7)	193 (3.5)	24 (10.3)
	9–12 years	3586 (61.5)	3421 (61.1)	165 (70.8)
	≥12 years	2027 (34.8)	1983 (35.4)	44 (18.9)
Hypertension	Yes, under treatment	2356 (40.4)	2243 (40.1)	113 (48.5)
	Yes, non‐treatment	223 (3.8)	217 (3.9)	6 (2.6)
Hyperlipidemia	Yes, under treatment	1526 (26.2)	1482 (26.5)	44 (18.9)
	Yes, non‐treatment	545 (9.4)	531 (9.5)	14 (6.0)
Diabetes	Yes, under treatment	555 (9.5)	520 (9.3)	35 (15.0)
	Yes, non‐treatment	164 (2.8)	158 (2.8)	6 (2.6)
Stroke	Yes	353 (6.1)	329 (5.9)	24 (10.3)
Drinking	Current	2595 (44.5)	2504 (44.7)	105 (45.1)
	Past	481 (8.3)	444 (7.9)	37 (15.9)
	Never	2754 (47.2)	2649 (47.3)	91 (39.1)
Smoking	Current	526 (9.0)	509 (9.1)	17 (7.3)
	Past	1488 (25.5)	1415 (25.3)	73 (31.3)
	Never	3816 (65.5)	3673 (65.6)	143 (61.4)
Frequency of going	Every day	4501 (77.2)	4343 (77.6)	158 (67.8)
outdoors	3–6 days/week	1150 (19.7)	1093 (19.5)	57 (24.5)
	≤2 days/week	179 (3.1)	161 (2.9)	18 (7.7)
Visual impairment	Yes	255 (4.4)	226 (4.0)	29 (12.5)
Hearing impairment	Yes	381 (6.5)	347 (6.2)	34 (14.6)
Grip strength	Low	1257 (21.6)	1142 (20.4)	115 (49.4)
Gait speed	Slow	585 (10.0)	511 (9.1)	74 (31.8)

Low grip strength was defined as <18 kg for women and <28 kg for men. The cut‐off level for slow gait speed was <1.0 m/s for both sexes.

In the simple model, all three variables were included (Table 2). The base model comprised nine variables, while the enhanced model was constructed from 10 variables. In the enhanced model, two physical function measurements were added, while the frequency of going outdoors was eliminated.

**Table 2:** Model development for cognitive impairment screening

		Simple model			Base model			Enhanced model		
	Category	*B*	OR (95% CI)	Score	*B*	OR (95% CI)	Score	*B*	OR (95% CI)	Score
Age	75–84 years	0.99	2.69 (1.95–3.71)	2	0.99	2.69 (1.94–3.72)	2	0.73	2.07 (1.48–2.89)	2
	≥85 years	1.96	7.12 (4.41–11.49)	3	1.95	7.03 (4.33–11.41)	5	1.37	3.95 (2.35–6.64)	4
Sex	Women	−0.65	0.52 (0.39–0.69)	−1	−0.58	0.56 (0.42–0.75)	−1	−0.58	0.56 (0.42–0.75)	−2
Body mass index	<18.5 kg/m^2^	—	—	—	0.57	1.77 (1.15–2.71)	1	0.45	1.57 (1.01–2.43)	1
	≥25.0 kg/m^2^	—	—	—	—	Not selected	—	—	Not selected	—
Education	<9 years	1.25	3.49 (1.99–6.10)	2	1.13	3.08 (1.75–5.43)	3	1.05	2.86 (1.61–5.08)	3
	9–12 years	0.94	2.57 (1.81–3.65)	1	0.91	2.49 (1.75–3.54)	2	0.92	2.50 (1.75–3.58)	2
Hypertension	Yes, under treatment	—	—	—	—	Not selected	—	—	Not selected	—
	Yes, non‐treatment	—	—	—	—	Not selected	—	—	Not selected	—
Hyperlipidemia	Yes, under treatment	—	—	—	−0.52	0.59 (0.42–0.84)	−1	−0.47	0.63 (0.44–0.89)	−1
	Yes, non‐treatment	—	—	—	—	Not selected	—	—	Not selected	—
Diabetes	Yes, under treatment	—	—	—	0.52	1.68 (1.13–2.49)	1	0.43	1.53 (1.02–2.29)	1
	Yes, non‐treatment	—	—	—	—	Not selected	—	—	Not selected	—
Stroke	Yes	—	—	—	—	Not selected	—	—	Not selected	—
Drinking	Current	—	—	—	—	Not selected	—	—	Not selected	—
	Past	—	—	—	0.43	1.54 (1.05–2.27)	1	0.37	1.45 (0.98–2.15)	1
Smoking	Current	—	—	—	—	Not selected	—	—	Not selected	—
	Past	—	—	—	—	Not selected	—	—	Not selected	—
Frequency of going outdoors	3–6 days/week	—	—	—	—	Not selected	—	—	Not selected	—
	≤2 days/week	—	—	—	0.50	1.65 (0.96–2.84)	1	—	Not selected	—
Visual impairment	Yes	—	—	—	1.02	2.76 (1.78–4.29)	2	0.83	2.30 (1.47–3.61)	2
Hearing impairment	Yes	—	—	—	—	Not selected	—	—	Not selected	—
Grip strength	Low	—	—	—	—	—	—	0.69	1.99 (1.47–2.70)	2
Gait speed	Slow	—	—	—	—	—	—	0.86	2.37 (1.70–3.28)	2

*B*: unstandardized beta.

Low grip strength was defined as <18 kg for women and <28 kg for men. The cut‐off level for slow gait speed was <1.0 m/s for both sexes.

Reference categories: age: 65–74 years; sex: men; body mass index: 18.5–24.9 kg/m^2^; education: >12 years; medical histories: no; drinking: never; smoking: never; frequency of going outdoors: every day; visual impairment: no; hearing impairment: no; grip strength: normal; gait speed: normal.

The AUCs of the three models ranged from 0.750 to 0.811 (Table 3). The AUC of the base model was significantly higher than that of the simple model (*P* < 0.001). The 95% CI for the continuous NRI and IDI did not include the null value after adding the other variables that were handled as basic information in this study to the simple model. There was a significant difference between the AUC of the base and the enhanced model (*P* < 0.001). The 95% CI for the continuous NRI and IDI did not overlap the null value after adding grip strength and gait speed to the base model. The *P*‐value for the Hosmer–Lemeshow test was higher than 0.05 in the three models. The calibration plots for each model are shown in Figure S1. The calibration is acceptable for many participants, although an over‐estimation is observed when the predicted probability of the base and enhanced models is over roughly 0.3 when less than 5% of participants are included.

**Table 3:** Performance of developed models

	Simple model	Base model		Enhanced model
AUC (95% CI)	0.750 (0.721, 0.779)	0.778 (0.751, 0.805)		0.811 (0.786, 0.835)
*P*‐value for Hosmer–Lemeshow test	0.420	0.433		0.125
*P*‐value for AUC difference	—	<0.001†		<0.001^‡^
NRI	—	0.450 (0.292, 0.592)†		0.538 (0.283, 0.662)^‡^
IDI	—	0.015 (0.004, 0.033)†		0.010 (0.001, 0.026)^‡^

^†^ Comparison with the age and sex model.

^‡^ Comparison with the base model.

NRI, continuous net reclassification improvement; IDI, integrated discrimination improvement.

Table 4 shows the summarized results of the score‐based models. A reduction of the AUC in each model was found after we converted regression coefficients into integer scores. The sensitivity was approximately 70% in the three score‐based models, and the specificity gradually increased along with the improvement of models.

**Table 4:** Performance of score‐based models

	Score‐based simple model	Score‐based base model	Score‐based enhanced model
AUC (95% CI)	0.721 (0.691, 0.750)	0.758 (0.730, 0.786)	0.792 (0.766, 0.818)
Cut‐off score	1/2	2/3	2/3
Sensitivity, % (95% CI)	71.7 (65.4, 77.4)	70.0 (63.6, 75.8)	72.5 (66.3, 78.2)
Specificity, % (95% CI)	61.2 (60.0, 62.5)	67.0 (65.8, 68.2)	70.2 (69.0, 71.4)

## Discussion

Numerous cognitive measurements with various features have been developed, and suitable measurement tools are used depending on the context in which cognitive assessments are conducted. Given this background, we developed models that can be utilized in community settings to screen older adults with cognitive impairment. The models were constructed using 10 or fewer variables without specific measurements such as blood tests and had acceptable discrimination and calibration, particularly the base and enhanced models.

In the present study, the AUC of all score‐based models was over 0.7, meaning that these models have acceptable discrimination.^20^ In previous cross‐sectional studies using the same cut‐off point for the MMSE, the AUC was 0.65 when the model included the specific motor‐cognitive dual‐task named Stepping Trail Making Test with covariates such as age, sex, education, and BMI,^7^ and was 0.85 when the programmed cognitive test called Comp‐Based CAT was carried out using a computer.^5^ Regarding the discrimination, the score‐based models developed by us are, to some extent, inferior to tests directly assessing cognitive function, such as the Comp‐Based CAT. Although neither our models nor such computer‐based assessments need expert knowledge for their evaluation of cognitive function, our models are easy to conduct since most parts comprise questionnaire‐based items even in the enhanced model. Our models, therefore, could be useful in situations where a simple cognitive screening is required. We found that the 95% CI for continuous NRI and IDI did not contain the null in either analysis (Table 3), which is equivalent to *P* < 0.05. These results suggest that it would be better to use at least the base model and, if possible, grip strength and gait speed should be added to improve reclassification ability.

For the base and the enhanced models, the selected variables were entirely reasonable since we decided on candidate variables based on previous findings.^11^, ^12^ Regarding sex, women showed significantly lower odds ratios relative to men. This finding appears to be incongruous with the results from meta‐analyses that show that the prevalence of dementia in women is higher than that in men^21^ and that there is no sex difference in the prevalence of mild cognitive impairment.^22^ It is difficult to make sense of why such discrepancies were observed. A possible reason for the inconsistent results is that the association between gender and cognitive function may have been emphasized because potential confounders, such as depressive symptoms and accessibility to where the field surveys were conducted, were not controlled for. In addition, given the MMSE scores of participants with cognitive impairment, most of these participants may not have had severe cognitive impairment (i.e., their stage might be possible/probable dementia or equivalent to mild cognitive impairment, but not moderate/severe dementia). Regarding the studies included in a meta‐analysis, some reported that women were less likely to have mild cognitive impairment than men, whereas others reported the opposite.^22^ Thus, it is possible that the results regarding gender effects are not unique to the sample in this study. Nevertheless, further studies are needed to examine gender differences in risk factors of cognitive impairment and dementia.^23^

Participants with hyperlipidemia who received treatment had a lower odds ratio compared with those without it. Those with hyperlipidemia are often prescribed statins, which could reduce the risk of mild cognitive impairment and dementia. A previous meta‐analysis showed that the use of statins is associated with a 26% lower relative risk of mild cognitive impairment and with a 15% lower relative risk of all‐cause dementia.^24^ However, it is important to interpret this possibility carefully. Since we did not have any data on medication use, a detailed analysis is needed to compare the data of individuals with hyperlipidemia who are prescribed statins with the data of those who are not.

A strength of this study is the sample size, which enabled us to perform logistic regression analysis stably even though event occurrence (the proportion of participants with cognitive impairment) was less than 5%.^25^ However, this study has several limitations. First, this study includes selection bias, which probably originates from the difference of sampling methods in each cohort included in the IRIDE‐CS. Consequently, the prevalence of cognitive impairment (i.e., MMSE < 24) was relatively low, although a similar case that showed the prevalence of MMSE < 24 was less than 5% was reported.^26^ Second, we missed some variables associated with cognitive function (e.g., depressive symptoms, other diseases such as cancer and osteoporosis, leisure time activities such as physical and cognitive activities), as we could not integrate or obtain relevant data. Further studies are needed to examine the extent to which these factors improve the discrimination of screening models. In this context, given that using a standardized questionnaire with several items to accurately assess one factor (e.g., depressive symptoms and amount of physical activity) reduces the practicality of the models, future studies should investigate ways to develop screening models including these factors. In addition, it might be helpful to examine the amount of alcohol consumption and tobacco smoking, but in this study we did not. These references may accumulate evidence on factors that must be incorporated into screening models, which might help in the development of sophisticated models. Third, the scope of application of the developed models is limited. The calibration plots show that the predicted probabilities diverge from observed probabilities; thus, caution is needed when the models are used to screen for cognitive impairment in older adults with extremely high scores. However, it would be rational to consider those with extremely high scores as having a lower cognitive function. Finally, we did not examine the external validity of the developed models, which should be addressed in future studies. Related to this, our findings may depend on the characteristics of the MMSE. The associations between the scores on the models we developed and other cognitive test scores should be explored in the future.

In conclusion, we have developed screening models for cognitive impairment using integrated data and created score‐based models. The score‐based base and enhanced models in particular are easy to use and have good discrimination. These features help in the screening of older adults with cognitive impairment in community settings, particularly when it is difficult to conduct a detailed cognitive assessment. Given the sensitivity and specificity of these models, it would be better to conduct standardized cognitive tests in clinical settings when older adults have scores close to or over the cut‐off point.

## Disclosure statement

The authors declare no conflict of interest.

## Supporting information


**Appendix** S1 List of the IRIDE Cohort Study investigators

**Figure S1** Calibration plots comparing observed frequencies with predicted probabilities

Table S1 Brief summary of each cohort study

## Data Availability

Research data are not shared.
