# Supplementary material for: Simple screening models for cognitive impairment in community settings: The IRIDE Cohort Study
Source: Geriatr Gerontol Int. 2022 Feb 20;22(4):292–7. doi: 10.1111/ggi.14360 (PMC9306945; doi:10.1111/ggi.14360)
Supplement: Supplementary file 1 — Appendix S1 List of the IRIDE Cohort Study investigators [file GGI-22-292-s001.docx]

Supplemental list. List of the IRIDE Cohort Study investigators

**IRIDE cohort study**

Kenji Toba, Shuichi Awata, Yoshinori Fujiwara, Tatsuro Ishizaki, Hunkyung Kim, Shuichi P. Obuchi, Takumi Abe, Mari Yamashita

**Otassha Study**

Otasha study 2011: Shuichi P. Obuchi*, Hisashi Kawai^†^, Hirohiko Hirano^†^, Hunkyung Kim^†^, Yoshinori Fujiwara^†^, Kazushige Ihara^†^, Manami Ejiri^†^, Yuki Ohara, Keiko Motokawa, Maki Shirobe, Masanori Iwasaki, Hiroyuki Suzuki, Masahiro Hashizume, Mitsugu Hachisu, Yutaka Watanabe, Motonaga Kojima, Takeshi Kera, Akiko Miki, Takahashi Junta, Kumiko Ito

Otasha study 2017&2019: Hunkyung Kim*, Hiroyuki Sasai^†^, Narumi Kojima^†^, Yosuke Osuka^†^

**Takashimadara Study**

Shuichi Awata*, Hunkyung Kim, Hirohiko Hirano, Tsuyoshi Okamura, Yuki Ohara, Hiroki Inagaki, Chiaki Ura, Ayako Edahiro, Naoko Sakuma, Mika Sugiyama, Fumiko Miyamae, Narumi Kojima, Yosuke Osuka, Keiko Motokawa, Hiroyuki Suzuki, Hidenori Amano, Satoshi Seino, Ryota Sakurai, Yukie Masui, Hisashi Kawai, Shoji Shinkai, Yutaka Watanabe, Akihiko Kitamura, Chiho Shimada, Madoka Ogawa, Yu Taniguchi

**SONIC study**

Tatsuro Ishizaki*, Yukie Masui^‡^, Yasuyuki Gondo^‡^, Kazunori Ikebe^‡^, Kei Kamide^‡^, Yasumichi Arai^‡^, Yuri Miura, Hiroki Inagaki, Madoka Ogawa, Yuko Yoshida, Saori Yasumoto, Ayaka Kasuga, Kodai Hatta, Toshihito Takahashi, Masahiro Kitamura, Shinya Murakami, Mai Kabayama, Kayo Godai, Hiroshi Akasaka, Hiromi Rakugi, Yoshiko Ishioka, Takeshi Nakagawa, Hiroyuki Muto

**Hatoyama Study**

Yoshinori Fujiwara*, Akihiko Kitamura^‡^, Shoji Shinkai^‡^, Mariko Nishi, Hiroshi Murayama, Yuri Yokoyama, Satoshi Seino, Yu Nofuji, Hidenori Amano, Miki Narita, Takumi Abe

**Kusatsu Longitudinal Study on Aging**

Yoshinori Fujiwara*, Akihiko Kitamura^‡^, Shoji Shinkai^‡^, Yu Nofuji, Takumi Abe, Hidenori Amano, Toshiki Hata, Hirohiko Hirano, Ai Iizuka, Tomoko Ikeuchi, Hiroshi Murayama, Miki Narita, Mariko Nishi, Yuki Ohara, Ryota Sakurai, Satoshi Seino, Hiroyuki Suzuki, Yu Taniguchi, Mari Yamashita, Yuri Yokoyama

* Principal investigator

^†^ Core member

^‡^ Co-principal investigator
