# Supplementary material for: Simple screening models for cognitive impairment in community settings: The IRIDE Cohort Study
Source: Geriatr Gerontol Int. 2022 Feb 20;22(4):292–7. doi: 10.1111/ggi.14360 (PMC9306945; doi:10.1111/ggi.14360)
Supplement: Supplementary file 2 — Figure S1 Calibration plots comparing observed frequencies with predicted probabilities [file GGI-22-292-s002.docx]

A) Simple model

B) Base model

C) Enhanced model

Supplementary Figure 1. Calibration plots comparing observed frequencies with predicted probabilities
