# Supplementary material for: Simple screening models for cognitive impairment in community settings: The IRIDE Cohort Study
Source: Geriatr Gerontol Int. 2022 Feb 20;22(4):292–7. doi: 10.1111/ggi.14360 (PMC9306945; doi:10.1111/ggi.14360)
Supplement: Supplementary file 3 — Table S1 Brief summary of each cohort study [file GGI-22-292-s003.docx]

Supplementary Table 1. Brief summary of each cohort study

| Cohort name | Sample size ^a^ | Period | Data provision for the IRIDE-CS (year) | Note |
| --- | --- | --- | --- | --- |
| Otassha Study | 3332  (≥65 years) | Ongoing study started in 2001. | 2015–2019 | The Otassha Study comprises the following three sub cohorts:  Otassha study 2011: This study’s participants were recruited from all residents aged 65–84 years living in nine residential areas in Itabashi ward, Tokyo, Japan. After excluding people who were institutionalized, 6,699 invitations for comprehensive health checkups were sent out in 2011.^1^ This baseline survey included 913 individuals. Past participants and new 65-year-old participants have been invited to follow-up surveys, approximately 800 people undergo the checkups every year. |
|  |  |  |  | Otassha 2017 cohort: The participants in this cohort were recruited from women aged 65–81 years living near the Tokyo Metropolitan Institute of Gerontology using the Basic Resident Register in 2017 (n = 6,788). After excluding 422 women who had participated in another cohort study, we sent out invitation letters to 6,366 candidates. Of them, 1,365 women (1,035 in 2017; 330 in 2018) completed the baseline survey. They are invited annually to participate in the follow-up survey.  Otassha 2019 cohort: The participants in this cohort were recruited from older adults aged 75–86 years living near the Tokyo Metropolitan Institute of Gerontology using the Basic Resident Register in 2019 (n = 4,233). After excluding 89 individuals who had participated in another cohort study, we sent out invitation letters to 4,144 candidates. Of them, 756 older adults (276 men and 480 women) completed the baseline survey in 2019. They have been invited annually to participate in the follow-up survey. |
| Takashimadaira Study | 2053  (≥70 years) | Ongoing study started in 2016. | 2016 | Takashimadaira study comprised three stages.  The 1st survey: We mailed self-administered questionnaires to 7,614 elderly persons aged 70 years or more living in districts one to five of Takashimadaira, Itabashi Ward, and collected 5,430 questionnaires (71.3%) using the mail-and-collect method.  The 2nd survey: Of the 5,430 people who participated in the first stage, 1,360 (17.9%) participated in a health checkup survey at a central location (central location survey) and 693 (9.1%) participated in an in-home survey (home visit survey).  The 3rd survey: We medically diagnosed the elderly persons suspected of impaired cognitive function in the second stage screening (MMSE <24). Of the 398 persons identified, 233 (3.1%) participated in this part of the survey. |
| SONIC study | 567  (≥70 years) | Ongoing study started in 2010. | 2011–2013 | The SONIC survey started in 2010 and is conducted in four regions of Kansai and Kanto in Japan.^2^ Participants were recruited from all those who lived in four regions and were 70±1, 80±1, and 90±1 years old at the time of the baseline survey. The number of participants in the baseline survey was 3,346. These participants have been followed up with once every three to four years. All participants underwent the Montreal Cognitive Assessment to assess cognitive function, and one-sixth of them were randomly selected to do the MMSE. |
| Hatoyama Cohort Study | 742  (≥65 years) | 2010–2018 | 2010 | The Hatoyama cohort was conducted in Hatoyama town, Saitama Prefecture, Japan. Follow-up surveys were conducted every two years. Participants were recruited using a stratified sampling method considering age (65–74 and 75–84 years) and residential area at baseline in 2010. The study profile has been published elsewhere.^3^ |
| Kusatsu Longitudinal Study on Aging | 1139  (≥65 years) | Ongoing study started in 2001. | 2010–2016 | The target population of this study are all residents aged 65 years and older in Kusatsu town, Gunma Prefecture, Japan. This study has been conducted every year since 2001.^4^ All residents aged 65 and older can participate in the study, and a cumulative total of over 10,000 participants have participated in the study. The initial data were extracted if those participants attended the study two or more times from 2010 to 2016. The study period was decided considering the data management policy of the organized group for the study. |

^a^: The number of participants involved in the IRIDE-CS study.

1. Fujiwara Y, Suzuki H, Kawai H, et al. Physical and sociopsychological characteristics of older community residents with mild cognitive impairment as assessed by the Japanese version of the Montreal Cognitive Assessment. *J Geriatr Psychiatry Neurol* 2013; 26: 209-220.

2. Gondo Y, Masui Y, Kamide K, et al. SONIC study: a longitudinal cohort study of the older people as part of a centenarian study. In: Pachana NA eds. *Encyclopedia of Geropsychology*. Singapore: Springer Science+ Business Media, 2016.

3. Murayama H, Nishi M, Shimizu Y, et al. The Hatoyama Cohort Study: design and profile of participants at baseline. *J Epidemiol* 2012; 22(6): 551-558.

4. Shinkai S, Yoshida H, Taniguchi Y, et al. Public health approach to preventing frailty in the community and its effect on healthy aging in Japan. *Geriatr Gerontol Int* 2016; 16: 87-97.
